# Associations of sleep duration and fruit and vegetable intake with the risk of metabolic syndrome in Chinese adults

**DOI:** 10.1097/MD.0000000000024600

**Published:** 2021-03-12

**Authors:** Ming-hui Wang, Tong Shi, Qiang Li, Hong-Mei Chen, Ming-wei Liu, Yuan-an Lu, Qiqiang He, Rui Chen

**Affiliations:** a School of Health Sciences; b Center for Population and Health Research, Wuhan University; c School of Nursing, Wuchang University of Technology, Wuhan, China; d Environmental Health Laboratory, Department of Public Health Sciences, University Hawaii at Manoa, Honolulu, USA.

**Keywords:** Chinese adults, fruit and vegetable intake, metabolic syndrome, Sleep duration

## Abstract

To understand the adverse association of short sleep duration and insufficient fruit and vegetable intake (FVI) with and their combined effect on metabolic syndrome (MetS) in Chinese adults.

This cross-sectional study analyzed 7052 adults aged 18∼64 years old in 2009, with fasting blood samples collected. Participants were divided into short/normal/long sleep duration groups and sufficient/insufficient FVI groups in accordance with self-reported information. Metabolic syndrome was defined by National Cholesterol Education Program's Adult Treatment Panel III criteria.

The prevalence of MetS among the study subjects was 21.74%. Participants were classified into short (<7 h/d), normal (7∼9 h/d), and long (>9 h/d) groups according to their daily sleep duration. Participants with less than 500 g of FVI per day was considered as insufficient FVI. After adjusting for confounders, the negative effect of short sleep duration on MetS was statistically significant, with an OR of 1.29 (95%CI = 1.06∼1.56); and high fasting glucose levels were significantly associated with insufficient FVI. Compared with subjects with normal sleep duration and sufficient FVI, participants with short sleep time and insufficient FVI had the highest risk of MetS (OR = 1.37, 95% CI: 1.04–1.66).

This study revealed that insufficient FVI and short sleep duration were significantly associated with an increased risk of MetS among Chinese adults. Increasing FVI and normal sleep duration during Chinese adults could be significant targets for reducing the prevalence of MetS.

## 1 Introduction

Metabolic Syndrome (MetS) is a cluster of illnesses that include central obesity, dyslipidemia, insulin resistance, glucose intolerance, hypertension, and endothelial dysfunction.^[1]^ It is estimated that the prevalence of MetS is between 20% to 30% in adults globally.^[2]^ Metabolic syndrome is associated with a pro-oxidant, pro-inflammatory, and pro-thrombotic state,^[3]^ and significantly contributed to the increased risk of cardiovascular diseases and type 2 diabetes mellitus. Thus, it is essential to prevent MetS and lessen the risk of related metabolic disorders.

Unhealthy lifestyles including unbalanced dietary and poor sleep have been recognized as adverse factors for MetS, but with inconsistent results. Some research showed an inverse association between fruit and vegetable intake (FVI) and MetS,^[4–6]^ while a meta-analysis of eight randomized controlled trials manifested that high FVI had no function on improving the MetS conditions among patients^[7]^ Homoplastically, conflicting findings were reported regarding the association between sleep duration and MetS. One meta-analysis described a U-shaped association between extremes of sleep duration and MetS,^[8]^ while another study did not show any significant association between long sleep duration and MetS.^[9]^

In China, the prevalence of MetS has nearly doubled in the last decade, and is expected to continue to rise because of the changes of lifestyle.^[10,11]^ A remarkable decrease in FVI was documented among Chinese adults, and the ratio of adults who met the Chinese dietary guidelines of daily FVI reduced from 58.8% in 1993 to 50.2% in 2011.^[12]^ A study found that about 11% of adults suffered from insufficient sleep in China.^[13]^ Current studies on the combined associations between sleep duration, FVI and MetS were limited. Therefore, this study aimed to explore the effects of sleep duration and FVI on the risk of MetS among Chinese adults.

## 2 Method

### 2.1 Study population

The China Health and Nutrition Survey (CHNS) is an ongoing and longitudinal study with samples from nine different provinces (Hunan, Jiangsu, Liaoning, Heilongjiang, Hubei, Henan, Guangxi, Shandong, Guizhou) throughout China from 1989 to 2015. The focus of the CHNS was to examine a series of economic, sociological, demographic and health questions. Details about the study design and sampling strategies are available elsewhere.^[14]^

The CHNS began to include blood samples in 2009, which allowed researchers to assess metabolic health outcomes. The sample was drawn from a multi-stage random cluster sampling process. All participants signed written informed consent. The study was approved by the China Center for Disease Control and Prevention and the institutional review board from the University of North Carolina-Chapel Hill.

Our analysis included a wave of survey data collected in 2009. A total of 8581 adults aged 18 to 64 years with fasting blood samples were qualified for inclusion to this study. Among these subjects, 1378 individuals were excluded because of lacking anthropometric data including height, weight, and waist circumference, 44 participants were removed due to lacking sleep information, and 107 were removed because of lacking FVI information. Thus, a total of 7052 subjects were included in this analysis.

### 2.2 Measurements

Weight, height and waist circumference were measured for all participants by trained examiners. Body mass index (BMI, kg/m^2^) was calculated from height and weight, and blood pressure (BP) was measured by educated interviewers using a mercury manometer. All blood samples were sent to a national central lab in Beijing (medical laboratory accreditation certificate ISO15189:2007) for processing. Biochemical markers including high-density lipoprotein (HDL) cholesterol, low-density lipoprotein cholesterol, total cholesterol, triglyceride (TG), and fasting plasma glucose (FPG) were analyzed in the China-Japan Friendship Hospital. Full laboratory analysis methods used for the biomarkers can be found elsewhere.^[15]^

Sleep duration was evaluated by a self-reported questionnaire. The question on sleep duration was asked by: “How many hours each day do you usually sleep, including during both daytime and nighttime?.” Participants were classified into short (<7 h/d), normal (7∼9 h/d), and long (>9 h/d) groups according to their daily sleep duration.^[16]^

Fruit and vegetable intake information was collected through 3 consecutive 24 hour recalls. Participants recorded the exact type and amount of foods they consumed during three consecutive days.^[17]^ Fruits and vegetables included in this analysis were based upon the classification of the “China Food Composition,” which classified daily food and their nutrition components.^[18]^ According to the Chinese dietary guidelines, participants with less than 500 g of FVI per day was considered as insufficient FVI.^[19]^

### 2.3 Covariates

Potential confounding factors including age, gender, residence, physical activity, alcohol drinking, current smoking, and education level were self-reported by participants using interviewer-administered household questionnaires. Participants’ residence was categorized as residing in rural or urban areas. Educational level was divided into three groups: elementary school or below, secondary school, and senior high school or above. Physical activity was measured by multiplying the weekly time spent in each activity, and metabolic equivalent score was used for measuring the intensity of specific activity.^[20]^ We adopted the leisure physical activity domain defined in the sleep, leisure, occupation, transportation and home model.^[21]^ This study contained 13 items of active and sedentary leisure physical activity. The amount of fibre for each food was obtained from the Chinese Food Composition Table. Individual daily intake value for each food item was provided by the dietary data. Per capita daily fibre was calculated by combining the amount of fibre for each food and individual daily intake value for each food item.^[22]^

### 2.4 Metabolic syndrome

According to the updated Adult Treatment Panel III of the National Cholesterol Education Program, MetS was defined as presenting with three or more of the following 5 ingredients:

(1) Abdominal obesity: waist circumference ≥90 cm in men or ≥80 cm in women;
(2) Hypertriglyceridemia: TG≥1.7 mmol/L or antihypertensive medication;
(3) Low HDL: HDL< 1.03mmol/L in men or 1.3 mmol/L in women;
(4) hypertension: BP≥130/85 mmHg or antihypertensive treatment;
(5) High fasting glucose: FPG≥6.1 mmol/L or previously diagnosed type 2 diabetes.^[23]^

### 2.5 Data analyses

Basic characteristics of participants were examined by analysis of covariance for continuous variables, and Chi-squared test was conducted for categorical variables, separately. An interaction test was performed to evaluate the interactive effect of sleep duration and FVI on the risk for MetS. All participants were divided into 6 groups based on their sleep duration and FVI level, and the combined effects of sleep duration and FVI on MetS were examined. Multivariable logistic regression was used to explore the association between sleep duration, FVI and MetS, after adjusting for gender, age, residence area, alcohol use, smoking status, physical activity, educational level, and energy intake. All statistical analyses were 2-tailed, and we considered differences as significant at *P* <.05. SPSS software (version 13.0; SPSS Inc.) was used for all analyses.

## 3 Results

Table 1 summarizes the descriptive characteristics of the study population. The average age of participants was 45.7 ± 11.9 years, 67.8% of respondents resided in rural areas. 31.0% smoked and 34.8% drank alcohol. A total of 21.7% of participants had MetS, with 20.0% in males and 23.3% in females, respectively. 24.9% of participants ate adequate fruit and vegetables daily, and 80.8% of participants had normal sleep duration.

**Table 1:** Basic characteristics of the study population.

	Total (n = 7052)	Men (n = 3305)	Woman (n = 3747)	*P* value
Age (years)	45.7 ± 11.9	45.7 ± 12.1	45.7 ± 11.8	.924
BMI (kg/m^2^)	23.4 ± 3.4	23.4 ± 3.4	23.4 ± 3.5	.839
Rural area (%)	4780 (67.78)	2245 (67.93)	2535 (67.65)	.818
Smoke (%)	2190 (31.03)	2068 (62.55)	122 (3.23)	<.001
Alcohol intake (%)	2454 (34.78)	2109 (63.81)	345 (9.16)	<.001
Education level (%)				.002
Low	4013 (68.8)	1988 (67.00)	2025 (70.66)	
Middle	1446 (24.79)	762 (25.68)	684 (23.87)	
High	374 (6.41)	217 (7.31)	157 (5.48)	
Physical activity (met-h/week)	7.4 ± 7.7	8.2 ± 8.9	6.7 ± 6.4	<.001
Energy intake (kcal/day)	2184.2 ± 664.2	2388.0 ± 664.0	2004.8 ± 611.8	<.001
SBP (mmHg)	122.3 ± 17.5	124.1 ± 16.1	120.6 ± 18.6	<.001
DBP (mmHg)	80.1 ± 11.2	82.0 ± 10.9	78.5 ± 11.3	<.001
TG (mmol/L)	1.7 ± 1.5	1.9 ± 1.8	1.5 ± 1.3	<.001
HDL (mmol/L)	1.4 ± 0.5	1.4 ± 0.5	1.5 ± 0.5	<.001
FPG (mmol/L)	5.3 ± 1.4	5.4 ± 1.6	5.3 ± 1.3	<.001
Total meat intake (g/day)	133.9 ± 111.2	148.0 ± 119.1	121.5 ± 103.3	<.001
Fibre intake (g/day)	12.8 ± 8.6	13.3 ± 8.4	12.3 ± 8.7	<.001
Fat intake (g/day)	76.1 ± 46.2	81.5 ± 45.5	71.3 ± 40.2	<.001
Sufficient FVI (%)	1754 (24.87)	846 (25.60)	908 (24.23)	.195
Normal sleep duration (%)	5698 (80.80)	2670 (80.79)	3028 (80.81)	.976
MetS (%)	1533 (21.74)	661 (20.00)	872 (23.27)	<.001

Values are mean ± SD for continuous variables and n (%) for categorical variables. BMI = body mass index, DBP = diastolic blood pressure, FPG = fasting plasma glucose, FV = fruits and vegetables, HDL = high-density lipoprotein, MetS = metabolic syndrome, SBP = systolic blood pressure, TG = triglycerides, WC = waist circumference.

Table 2 exhibits the prevalence of MetS and its ingredients among different sleep duration and FVI groups. Compared to those with normal sleep duration, participants with short sleep duration showed significantly higher BP, FPG, and increased proportion of MetS. However, no statistically significant difference was found between normal sleep duration group and long sleep duration group. On the other hand, participants with insufficient FVI tended to have higher levels of SBP, DBP, FPG, TG, and proportion of MetS, although only high fasting glucose levels were significantly associated with insufficient FVI.

**Table 2:** Odds ratio (95% confidence interval) for the metabolic syndrome components according to sleep duration and FVI.

	Normal sleep duration (n = 5698)	Short sleep duration (n = 670)	*P* value	Long sleep duration (n = 684)	*P* value	Sufficient FV (n = 1754)	Insufficient FV (n = 5298)	*P* value
MetS (%)	1200 (21.06)	189 (28.21)		144 (21.05)		379 (21.61)	1154 (21.78)	
MetS risk (OR, 95% CI)	Reference	1.29 (1.06∼1.56)	.021	0.92 (0.84∼1.27)	.228	Reference	1.06 (0.92∼1.23)	.487
MetS components								
Abdominal obesity (%)	2327 (40.84)	311 (46.42)		291 (42.54)		741 (42.25)	2188 (41.30)	
(OR, 95% CI)	Reference	1.14 (0.97∼1.37)	.273	1.07 (0.90∼1.27)	.890	Reference	0.99 (0.88∼1.11)	.778
High BP (%)	2085 (36.59)	296 (44.18)		231 (33.77)		651 (37.12)	1961 (37.01)	
(OR, 95% CI)	Reference	1.18 (1.00∼1.42)	.019	0.88 (0.75∼1.03)	.056	Reference	1.05 (0.92∼1.18)	.457
Hypertriglyceridemia (%)	1785 (31.33)	243 (36.27)		230 (33.63)		562 (32.04)	1696 (32.01)	
(OR, 95% CI)	Reference	1.19 (0.99∼1.42)	.215	1.12 (0.94∼1.33)	.766	Reference	1.02 (0.91∼1.15)	.785
Low HDL (%)	1493 (26.20)	167 (24.93)		177 (25.88)		442 (25.20)	1395 (26.33)	
(OR, 95% CI)	Reference	0.92 (0.77∼1.11)	.461	0.99 (0.83∼1.18)	.822	Reference	1.04 (0.91∼1.19)	.549
High fasting glucose (%)	678 (11.90)	103 (15.37)		70 (10.23)		189 (10.78)	662 (12.50)	
(OR, 95% CI)	Reference	1.29 (1.02∼1.63)	.013	0.86 (0.66∼1.12)	.054	Reference	1.24 (1.03∼1.48)	.024

DBP = diastolic blood pressure, FV = fruits and vegetables, FPG = fasting plasma glucose, HDL = high-density lipoprotein, MetS = metabolic syndrome, SBP = systolic blood pressure, TG = triglycerides, WC = waist circumference. ^∗^Adjusted for age, gender, area, alcohol use, smoking status, physical activity, education level, total meat, fat, fibre, and energy.

In the interaction test, the term representing the interaction between sleep duration and FVI was not a significant predictor of MetS (*P* = .477). The combined effects of sleep duration and FVI on MetS are shown in Table 3. Compared with those with sufficient FV and normal sleep duration, participants with insufficient FVI and short sleep duration had significantly increased risk of MetS (OR: 1.37, 95% CI: 1.04–1.66).

**Table 3:** Combined associations of FVI and sleep duration with metabolic syndrome.

	Sufficient fruit and vegetables intake		Insufficient fruit and vegetables intake	
Sleep duration	N (%)	OR (95% CI)	N (%)	OR (95% CI)
Normal	1418 (20.87)	Reference	3376 (21.12)	1.06 (0.89–1.18)
long	171 (22.22)	1.11 (0.79–1.62)	513 (20.66)	1.01 (0.79–1.31)
Short	165 (27.27)	1.25 (0.88–1.87)	505 (28.51)	1.37 (1.04–1.66)^∗^

Results were adjusted for age, gender, area, alcohol use, smoking status, physical activity, education level, total meat, fat, fiber and energy. FVI = fruit and vegetable intake.

∗ *P* < .05.

## 4 Discussion

This population-based study examined the impact of sleep duration and FVI on the risk of MetS among Chinese adults. Our results show that short sleep duration group showed a significantly increased risk for MetS as compared with normal sleep duration group, and insufficient FVI was associated with higher FPG. Furthermore, the combination of short sleep duration and insufficient FVI were associated with the highest risk of MetS.

Sleep is an active physiological state, and it is characterized by dynamic fluctuations in the central nervous system, respiration, hemodynamics, and metabolic factors. Sleep can affect pathophysiology and cardiovascular function in diverse ways.^[24]^ Our findings showing the negative effect of short sleep duration on MetS and its related components are consistent with previous reports.^[25]^ For instance, a cross-sectional study revealed a positive relationship between sleep deprivation and MetS.^[26]^ Individuals with short sleep duration were also found to have increased risks of obesity, hypertension, diabetes and dyslipidemia.^[27–29]^ Furthermore, an experimental study indicated that short sleep duration might promote weight gain and alter glucose metabolism.^[30]^ On the other hand, the evidence linking long sleep duration to MetS is mixed. A large scale cross-sectional study demonstrated that individuals with short sleep duration were more likely to suffer metabolic syndrome than those with long sleep duration,^[31]^ but a prospective study reported that the increased risk of type 2 diabetes was only observed with long-time sleepers.^[32]^ However, this study didn’t find any significant association between long sleep duration and MetS risk. Possible reasons for these discrepancies may be due to differences in the average age of the population, study design, and different definitions regarding sleep duration and MetS. Further prospective cohort studies with larger sample size are warranted to understand the relationship of sleep duration with MetS.

In the present study, participants with insufficient FVI tended to have a higher risk of MetS and several related components, and a significant association was observed between insufficient FVI and high FGP. A meta-analysis indicated that FVI might be inversely associated with the risk of MetS^[6]^ whereas findings from another meta-analysis of eight RCTs suggest that FVI was only linked with the decrease of DBP, but no effect on other MetS-related components.^[7]^ The diverse findings from FVI across studies may partly account for these inconsistent findings. It was reported that green vegetable rather than all types of vegetable was connected with a reduced risk of MetS.^[33]^ Furthermore, ways for cooking vegetables and different nutritional compositions of fruits and vegetables may also lead to the deviation of effects. Therefore, the association between FVI and MetS needs further study.

In addition, this study revealed that individuals with insufficient FVI and short sleep duration had the highest risk of MetS. The exact mechanisms of synergistic effects of short sleep duration and insufficient FVI on metabolic health have yet been elucidated. One possible explanation is that short sleep duration may cause changes in circulating levels of leptin and ghrelin, which would in turn increase appetite and calorie intake, reduce energy expenditure and impair glycemic control.^[34,35]^ On the other hand, fruit and vegetables comprise relatively lower energy and higher fibre, and insufficient FVI may lead to increase the overall energy content of daily dietary intake.^[36]^ Furthermore, insufficient FVI may increase blood sugar due to the lower glycemic index of fruit and vegetables.^[4]^ Since both short sleep duration and insufficient FVI have been proven to increase the risk of MetS through several pathways, it is biologically plausible that the combination of short sleep duration and insufficient FVI may play a synergic role in the development of metabolic diseases.

There are several limitations in this study. First, causal effects cannot be drawn between sleep duration, FVI and MetS because of the cross-sectional design. Second, information on dietary intake and sleep was self-reported, thus recall bias might exist. Last, this study was restricted to Chinese adults. Hence, our findings might not be generalizable to other population.

In summary, short sleep duration and insufficient FVI may be significant risk factors for MetS among Chinese adults. The results suggest that adequate sleep duration and enough FVI can help to reduce potential damage to the cardiovascular system and prevent the occurrence of MetS.

## Author contributions

**Conceptualization:** Qiqiang He, Rui Chen.

**Methodology:** Yuanan Lu.

**Software:** Qiang Li, Hong-Mei Chen, Mingwei Liu.

**Writing – original draft:** Minghui Wang.

**Writing – review & editing:** Tong Shi.
